# Survival analysis of stage II gastric cancer patients after D2 gastrectomy: a Chinese people-based research

**DOI:** 10.1186/s12876-021-01937-9

**Published:** 2021-10-07

**Authors:** Zi-Jian Deng, Run-Cong Nie, Jun Lu, Xi-Jie Chen, Jun Xiang, Chang-Ming Huang, Ying-Bo Chen, Jun-Sheng Peng, Shi Chen

**Affiliations:** 1 Guangdong Provincial Key Laboratory of Colorectal and Pelvic Floor Diseases, Guangdong Institute of Gastroenterology, The Sixth Affiliated Hospital, Sun Yat-Sen University, No 26, Yuancun Erheng Road, Tianhe District, Guangzhou, 510655 Guangdong People’s Republic of China; 2 Department of Gastric Surgery, The Sixth Affiliated Hospital, Sun Yat-Sen University, Guangzhou, 510655 People’s Republic of China; 3 Department of Gastric Surgery, Sun Yat-Sen University Cancer Center, State Key Laboratory of Oncology in South China, Collaborative Innovation Center for Cancer Medicine, Guangzhou, People’s Republic of China; 4 Department of Gastric Surgery, Fujian Medical University Union Hospital, No. 29 Xinquan Road, Fuzhou, 350001 People’s Republic of China

**Keywords:** Gastric cancer, Stage II, Prognosis, Adjuvant chemotherapy, Propensity score matching

## Abstract

**Objective:**

The benefit of adjuvant chemotherapy is still controversial for stage II gastric cancer patients. This study aims to identify prognostic factors to guide individualized treatment for stage II gastric cancer patients.

**Methods:**

We retrospectively reviewed 1121 stage II gastric cancer patients who underwent D2 radical gastrectomy from 2007 to 2017 in the Sixth Affiliated Hospital of Sun Yat-sen University, FuJian Medical School Affiliated Union Hospital and Sun Yat-sen University Cancer Center. Propensity score matching was used to ensure that the baseline data were balanced between the adjuvant chemotherapy group and surgery-only group. Kaplan–Meier survival and multivariate Cox regression analyses were carried out to identify independent prognostic factors.

**Results:**

In univariate analysis, after propensity score matching, age, tumor location, tumor size, CEA, T stage and N stage were associated with overall survival (OS). Multivariate analysis illustrated that age ≥ 60 years old, linitis plastica and T4 were independent risk factors for OS, but lower location and adjuvant chemotherapy were protective factors.

**Conclusion:**

Stage II gastric cancer patients with adverse prognostic factors (age ≥ 60, linitis plastica and T4) have poor prognosis. Adjuvant chemotherapy may be more beneficial for these patients.

## Introduction

Despite the rapid advances that continue to improve comprehensive therapy and screening methods, gastric cancer is still the fourth most common malignant tumor in the world (989,600 new cases per year) and the second leading cause of death among all malignant tumors (738,000 deaths annually) [1]. Approximately 60% of new cases occur in three eastern Asian countries: China, Japan and South Korea [2]. Although the incidence of gastric cancer has decreased worldwide since the 1950s, China still accounts for 42.6% of the global incidence and approximately 45% of gastric cancer-related deaths [3, 4].

In recent years, adjuvant chemotherapy has been widely used for stage II and III gastric cancer patients to improve the 5-year overall survival rate. The ACTS-GC study, a randomized phase III trial, showed that the overall survival rate at 5 years was 71.7% in the adjuvant group and 61.1% in the surgery-only group (HR [95%CI], 0.669 [0.540–0.828]) [5]. Another famous study in Asia, the CLASSIC study, also reported a hazard ratio for 5-year overall survival of 0.66 (95% CI, 0.51–0.85; *p* = 0.0015) for surgery and adjuvant chemotherapy with capecitabine and oxaliplatin for 6 months after a median follow-up of 62.4 months [6]. These results from randomized controlled trials provided hard evidence of the survival benefits associated with adjuvant chemotherapy. However, the subgroup analysis of 3-year disease-free survival in the CLASSIC study demonstrated that the improvement in the adjuvant chemotherapy group for stage II disease was not as evident as that for stage IIIa or IIIb disease. The 3-year disease-free survival rates for stage II disease were 85% (adjuvant chemotherapy group) vs 71% (surgery-only group), those for stage IIIa disease were 66% versus 51%, and those for stage IIIb disease were 61% versus 33% [7]. Some clinics considered that adjuvant chemotherapy for all stage II or III gastric cancer patients may be unnecessary or even harmful to some patients [8]. Furthermore, Choi et al. confirmed that stage II or III gastric cancer patients with high microsatellite instability might not benefit from adjuvant chemotherapy [9]. Some researchers have tried to use biomarkers to determine the necessity of chemotherapy, but all of these studies were retrospective, and the results were unsatisfactory.

Therefore, there is still no evaluation system for stage II gastric cancer patients to determine if adjuvant chemotherapy is needed. Thus, the purpose of this study is to confirm the role of adjuvant chemotherapy in the treatment of stage II gastric cancer and determine some prognostic factors for the establishment of a prediction model to guide individualized treatment for stage II gastric cancer.

## Materials and methods

### Patients

We retrospectively collected clinicopathological factors from 1389 gastric cancer patients from the Sixth Affiliated Hospital of Sun Yat-sen University, FuJian Medical School Affiliated Union Hospital and Sun Yat-sen University Cancer Center who underwent D2 radical gastrectomy alone or radical surgery followed by adjuvant chemotherapy. We applied inclusion and exclusion criteria to select eligible patients. The inclusion criteria were as follows: (1) pathologically confirmed stage II gastric adenocarcinoma (according to the 8th TNM staging system of the American Joint Committee on Cancer), (2) D2 radical gastrectomy, (3) enough clinicopathological factors to analyze, (4) no synchronized tumors, and (5) complete follow-up data. The exclusion criteria were as follows: (1) previous neoadjuvant chemotherapy, immunotherapy or radiotherapy, (2) missing important clinicopathological factor information, (3) incomplete follow-up information, (4) severe hepatic and renal insufficiency, and (5) age younger than 18 years. Finally, a total of 1121 patients with stage II gastric cancer were selected, with 805 (71.8%) patients in the adjuvant chemotherapy (AC) group and 316 (28.2%) in the surgery-only group.

### Variables

The continuous variables were turned into suitable categorical variables. The analyzed variables included age (< 60, ≥ 60 years old), sex (male, female), carcinoembryonic antigen (CEA) (< 5, ≥ 5 µg/L), carbohydrate antigen 19–9 (CA199) (< 37, ≥ 37 U/ml), hemoglobin (HB) (< 120, ≥ 120 g/L), primary tumor location (upper, middle, lower or linitis plastica), primary tumor size (< 5, ≥ 5 cm), T stage (T1/2/3, T4), N stage (negative, positive), tumor histology (mucinous adenocarcinoma or signet cell carcinoma), and adjuvant chemotherapy. The cut-off points for age and tumor size were classified by the “x-tile” program. The cut-off values of CEA, CA199 and HB were taken as the reference standards. T stage and N stage were identified by the postoperative pathological report. Poorly differentiated adenocarcinoma, including mucinous adenocarcinoma and signet cell carcinoma, were separated from gastric adenocarcinoma for the analysis.

### Statistical analysis

Descriptive statistics were used to summarize distributions of the variables, and the chi-square test was used to evaluate the baseline categorical variables. Then, age was used as a covariate for propensity score matching (PSM). After 1:2 propensity score matching, the baseline clinicopathologic characteristics and overall survival (OS) were analyzed. OS was calculated from the date of surgery to death from cancer-related causes. The Kaplan–Meier method was used to draw survival curves, and the differences among curves were compared by log-rank test. Next, the variables with a *p* value < 0.10 in univariate analysis were enrolled in multivariate analysis. Multivariate Cox regression was used to determine independent prognostic factors associated with overall survival, and the final prognostic factors were selected by the stepwise method. A two-sided *p* value < 0.05 was considered significant in all analyses. All analyses were carried out by SPSS v.22.0 (SPSS, Inc., Chicago, IL, USA).

### Propensity score matching

The patients were not randomly distributed into the adjuvant chemotherapy group or surgery-only group, which contributed to selection bias, so PSM was used to control selection bias and balance unbalanced covariates associated with the outcome. In this study, 1:2 nearest-neighbor matching for PSM without replacement was utilized. The caliper width was 0.05.

## Result

### Patient characteristics

The clinicopathological factors of both group stage II gastric cancer patients are shown in Table 1. Before propensity score matching, the distribution of age (*p* = 0.002) and signet ring cell carcinoma (*p* = 0.007) were evidently different between the two groups. Compared with the surgery-only group, the AC group had fewer patients aged ≥ 60 years (48.4% vs 58.9%) but more patients with signet ring cell carcinoma (18.0% vs 11.4%). Moreover, there were no differences in sex, CEA, CA199, HB, site of primary tumor, size of primary tumor, T stage, N stage or mucinous adenocarcinoma between groups. To avoid overmatching, we selected age as a covariate to estimate the propensity score because age was associated with OS (*p* < 0.001, Table2). After 1:2 PSM, all variates were balanced (*p* > 0.05) except for signet ring cell carcinoma (*p* = 0.038). In total, 948 patients were selected by PSM, including 632 patients with adjuvant chemotherapy and 316 patients without adjuvant chemotherapy.

**Table 1:** Characteristics of Stage II Gastric Cancer Patients before and after PSM

Characteristic	Before PSM (n = 1121)			After PSM (n = 948)		
	AC [n = 805] (%)	Surgery-only [n = 316] (%)	*p* value	AC [n = 632] (%)	Surgery-only [n = 316] (%)	*p* value
Age			0.002			1.000
< 60	415 (51.6)	130 (41.1)		260 (41.1)	130 (41.1)	
≧ 60	390 (48.4)	186 (58.9)		372 (58.9)	186 (58.9)	
Sex			0.102			0.255
Male	567 (70.4)	238 (75.3)		454 (71.8)	238 (75.3)	
Female	238 (29.6)	78 (24.7)		178 (28.2)	78 (24.7)	
Tumor location			0.143			0.296
Upper	236 (29.3)	106 (33.5)		201 (31.8)	106 (33.5)	
Middle	139 (17.3)	44 (24.0)		105 (16.6)	44 (13.9)	
Lower	383 (47.6)	155 (49.1)		290 (45.9)	155 (49.1)	
Linitis plastica	47 (5.8)	11 (3.5)		36 (5.7)	11 (3.5)	
Tumor size			0.638			0.849
< 5 cm	519 (64.5)	199 (63.0)		402 (63.6)	199 (63.0)	
≧ 5 cm	286 (35.5)	117 (37.0)		230 (36.4)	177 (37.0)	
SCA			0.007			0.038
Yes	145 (18.0)	36 (11.4)		104 (16.5)	36 (11.4)	
No	660 (82.0)	280 (88.6)		528 (83.5)	280 (88.6)	
MCA			0.193			0.240
Yes	69 (8.6)	35 (11.1)		55 (8.7)	35 (11.1)	
No	736 (91.4)	281 (88.9)		577 (91.3)	281 (88.9)	
CA199 (U/ml)			0.623			0.436
< 37	710 (88.2)	282 (89.2)		553 (87.5)	282 (89.2)	
≧ 37	95 (11.8)	34 (10.8)		79 (12.5)	34 (10.8)	
CEA (ng/ml)			0.627			0.951
< 5	677 (84.1)	262 (82.9)		525 (83.1)	262 (82.9)	
≧ 5	128 (15.9)	54 (17.1)		107 (16.9)	54 (17.1)	
HB (g/L)			0.297			0.166
< 120	289 (35.9)	124 (39.2)		219 (34.7)	124 (39.2)	
≧ 120	516 (64.1)	192 (60.8)		413 (65.3)	192 (60.8)	
T stage			0.086			0.088
T1/2/3	665 (82.6)	247 (78.2)		523 (82.8)	247 (78.2)	
T4	140 (17.4)	69 (21.8)		109 (17.2)	69 (21.8)	
N stage			0.141			0.215
Positive	424 (52.7)	151 (47.8)		329 (52.1)	151 (47.8)	
Negative	381 (47.3)	165 (52.2)		303 (47.9)	165 (52.2)	

SCA, signet ring cell carcinoma; MCA, mucinous adenocarcinoma; CEA, carcinoembryonic antigen; CA199, carbohydrate antigen 19-9

**Table 2:** Univariate Analysis of Factors Associated with Overall Survival before and after PSM

	Before PSM (n = 1121)		After PSM (n = 948)	
	Mean OS (month) [95% CI]	*p* value	Mean OS (month) [95% CI]	*p* value
Age		< 0.001		< 0.001
< 60	113.90 [110.23–117.58]		115.39 [111.33–119.44]	
≧ 60	91.26 [86.45–96.07]		91.24 [86.37–96.11]	
Sex		0.025		0.075
Male	100.44 [96.72–104.17]		98.80 [94.89–102.71]	
Female	110.05 [104.45–115.66]		108.50 [102.22–114.77]	
Tumor location		< 0.001		< 0.001
Upper	93.41 [88.55–98.28]		91.61 [86.30–96.93]	
Middle	88.83 [81.59–96.07]		88.87 [80.62–97.12]	
Lower	110.89 [106.79–114.98]		111.21 [106.80–115.63]	
Linitis plastica	63.26 [51.61–74.91]		62.28 [49.97–74.59]	
Tumor size		< 0.001		0.002
< 5 cm	109.01 [105.20–112.83]		107.99 [103.72–112.27]	
≧ 5 cm	95.76[90.16–101.37]		89.71 [84.49–94.93]	
SCA		0.164		0.122
Yes	111.02 [104.65–117.38]		111.35 [104.17–118.52]	
No	101.11 [97.61–104.60]		97.42 [93.83–101.02]	
MCA		0.740		0.622
Yes	89.21 [82.56–95.87]		89.67 [82.71–96.63]	
No	104.98 [101.57–108.40]		104.22 [100.48–107.96]	
CA199 (U/ml)		0.143		0.093
< 37	106.21 [102.86–109.56]		105.86 [102.22–109.50]	
≧ 37	82.17 [74.61–89.74]		80.08 [71.58–88.58]	
CEA (ng/ml)		0.014		0.010
< 5	106.67 [103.22–110.12]		106.31 [102.54–110.08]	
≧ 5	78.17 [72.40–83.93]		77.03 [70.80–83.25]	
HB (g/L)		0.206		0.140
< 120	99.08 [94.01–104.14]		97.49 [91.83–103.15]	
≧ 120	106.34 [102.36–110.32]		106.21 [101.91–110.50]	
T stage		0.009		0.013
T123	103.16 [99.89–106.43]		102.45 [98.81–106.09]	
T4	97.99 [91.00–104.97]		97.02 [89.34–104.70]	
N stage		0.006		0.035
Positive	106.01 [102.45–109.57]		104.76 [100.73–108.80]	
Negative	99.95 [94.99–104.90]		100.08 [94.77–105.40]	

OS, overall survival; SCA, signet ring cell carcinoma; MCA, mucinous adenocarcinoma; CEA, carcinoembryonic antigen; CA199, carbohydrate antigen 19-9

### Univariate survival analysis

**Fig.1:**
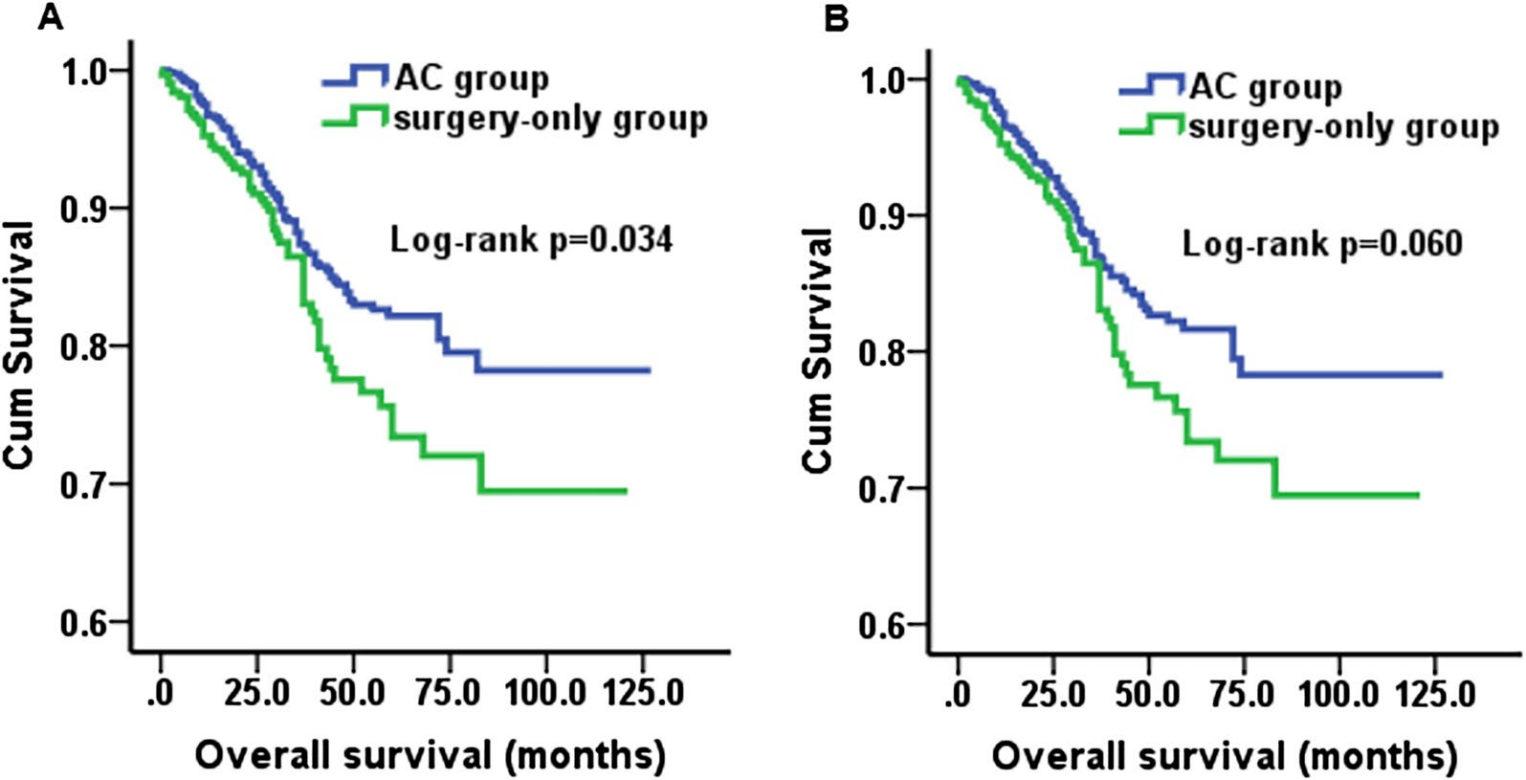
Kaplan–Meier analysis of overall survival in adjuvant chemotherapy group and surgery-only group. **A** Before PSM, *p* = 0.034; **B** after PAM, *p* = 0.060

The prognoses of the AC group and surgery-only group were compared by Kaplan–Meier survival analysis. Before PSM, the survival curves illustrated that the prognosis of the AC group was significantly better than that of the surgery-only group (*p* = 0.034). The mean overall survival time of the AC group was 107.48 ± 1.89 months, which was longer than that of the surgery-only group (95.53 ± 3.06 months). However, we amazingly found that after PSM, the difference in overall survival curves between the two groups was not as significant as before (*p* = 0.060). The mean overall survival time was 107.18 ± 2.12 months (AC) vs 95.53 ± 3.06 months (surgery-only) (Fig. 1).

**Fig. 2:**
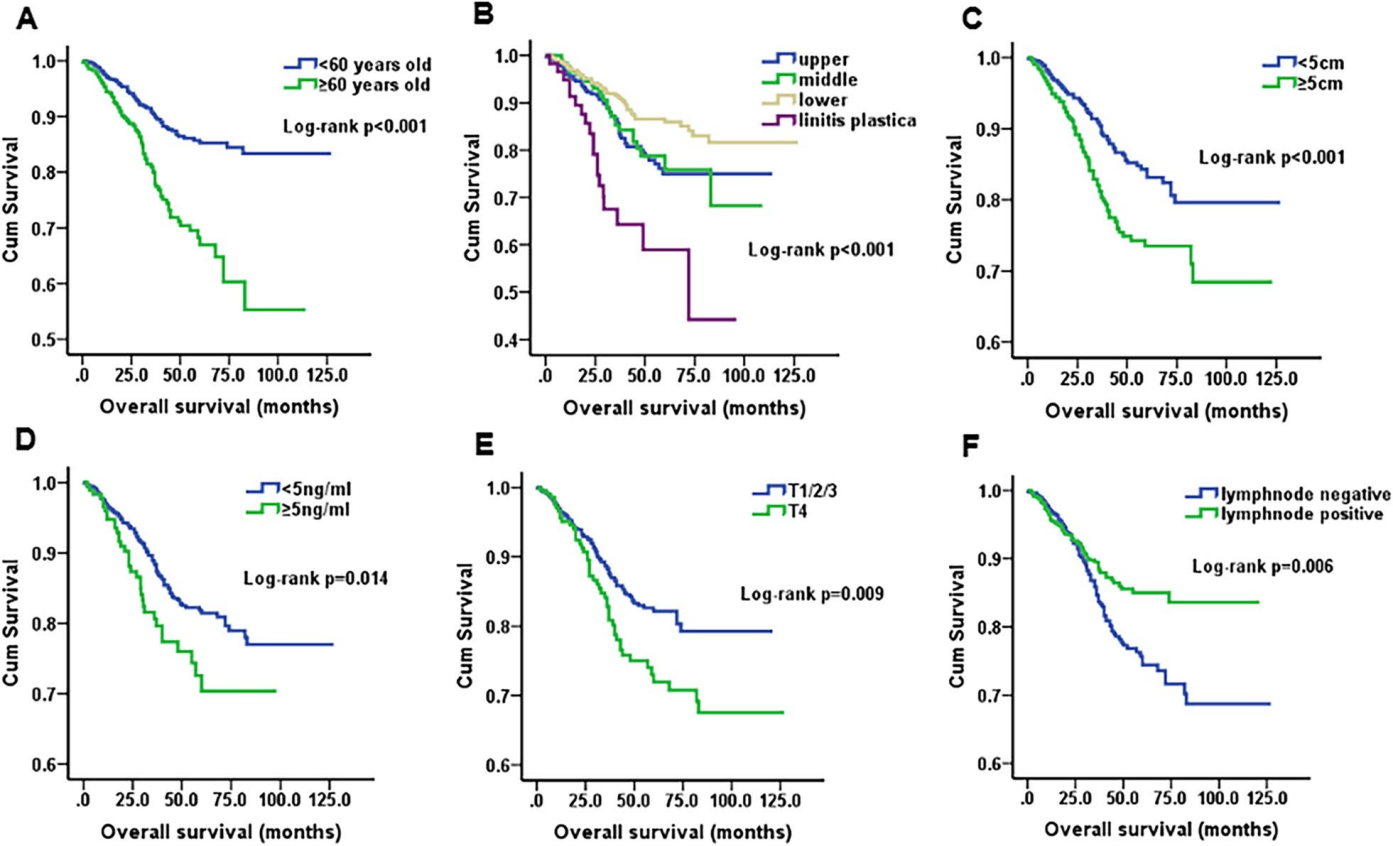
Kaplan–Meier survival curves of overall survival before PSM. **A** Age, *p* < 0.001; **B** Tumor location, *p* < 0.001; **C** Tumor size, *p* < 0.001; **D** CEA, *p* = 0.014; **E** T stage, *p* = 0.009; **F** N stage, *p* = 0.006

**Fig. 3:**
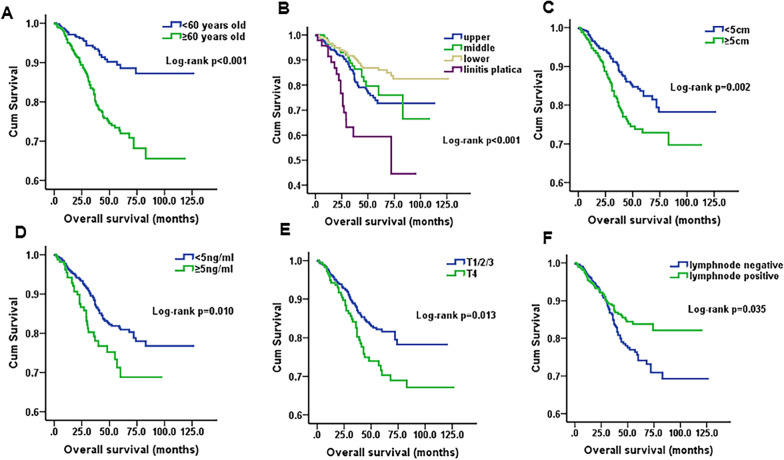
Kaplan–Meier survival curves of overall survival after PSM. **A** Age, *p* < 0.001; **B** Tumor location, *p* < 0.001; **C** Tumor size, *p* < 0.002; **D** CEA, *p* = 0.010; **E** T stage, *p* = 0.013; **F** N stage, *p* = 0.035

**Fig. 4:**
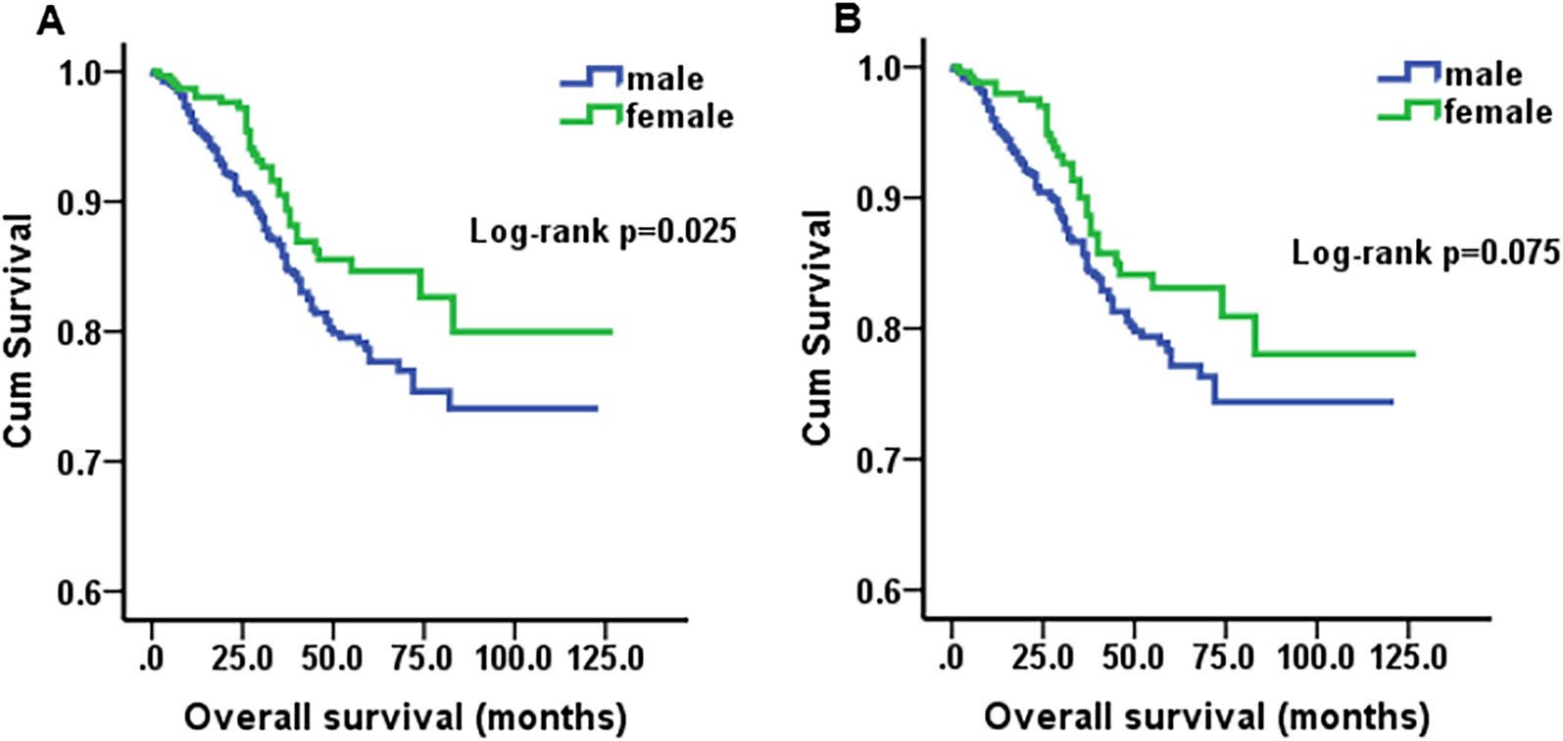
Kaplan–Meier survival curves of overall survival in sex. **A** Before PSM, *p* = 0.025; **B** after PSM, *p* = 0.075

Furthermore, the results also revealed that age (*p* < 0.001), primary tumor location (*p* < 0.001), primary tumor size (*p* < 0.001), CEA (*p* = 0.014), T stage (*p* = 0.009) and N stage (*p* = 0.006) were obviously associated with OS (Table 2, Fig. 2), but CA199 (*p* = 0.143), HB (*p* = 0.206), mucinous adenocarcinoma (*p* = 0.740) and signet ring cell carcinoma (*p* = 0.164) had statistically insignificant associations (Table 2). The results of the above variables were similar after PSM (Table 2, Fig. 3). Regarding sex, female patients had better survival than male patients before PSM (*p* = 0.025). However, the differences in OS between sexes were not significantly after PSM (*p* = 0.075) (Fig. 4).

### Multivariate Cox regression analyses

Cox proportional hazard models were established to identify independent prognostic factors for overall survival after matching. The results demonstrated that old age, high T stage and linitis plastica were independent risk factors for poor survival, while lower primary tumor and adjuvant chemotherapy were protective factors (Table 3).

**Table 3:** Multivariate Cox regression analysis of factors associated with overall survival after PSM

	Overall survival	
	HR [95%CI]	*p* value
Age		
< 60	Reference	
≧ 60	2.953 [1.959–4.452]	< 0.001
Tumor location		
Upper	Reference	
Middle	0.867 [0.523–1.437]	0.579
Lower	0.619 [0.416–0.920]	0.018
Linitis plastica	2.647 [1.528–4.585]	0.001
T stage		
T123	Reference	
T4	1.667 [1.148–2.421]	0.007
Adjuvant chemotherapy		
No	Reference	
Yes	0.683 [0.485–0.964]	0.030

*HR* hazard ratio, *CI* confidence interval

## Discussion

Although the incidence of gastric cancer is declining worldwide [10], most gastric cancer patients are diagnosed at an advanced stage in China, so D2 radical gastrectomy and adjuvant chemotherapy still play a critical role in gastric cancer treatment [4]. Many important clinical studies have confirmed the significance of adjuvant chemotherapy, including the INT-0116 study [11], MAGIC study [12], CLASSIC study [6, 7] and ACTS-GC study [5, 13]. Recently, JCOG1104 trail further revealed the overall survival of stage II gastric cancer patients with eight course adjuvant S1 was better than those with four courses, but T1N2-3 and T3N0 were excluded [14]. However, most patients in the INT-0116 study underwent D0 or D1 gastrectomy (a total of 90%). Likewise, D2 radical gastrectomy was not used as standard surgery in the MAGIC study. Therefore, some Asian doctors doubt that these results are appropriate for gastric cancer patients because D2 radical gastrectomy has been the standard of care in east Asia [15, 16]. In addition, the CLASSIC study and ACTS-GC study have confirmed the positive effects of adjuvant chemotherapy after D2 gastrectomy, but their inclusion criteria were based on the 6th edition AJCC and 2nd edition Japanese Gastric Cancer Association (JGCA) guidelines, respectively. Compared with 6th edition AJCC gastric cancer TNM staging, the most significant differences in the 8th edition AJCC used in our study was that the tumor with subserosa invasion was defined as T3 instead of T2. In addition, 3–6 regional lymph nodes metastasis were redefined as N2 instead of N1, 7–15 regional lymph nodes metastasis was N3a and ≥ 16 was N3b. Thus, T3N2 were excluded from stage II gastric cancer and T1N2 and T3N0 were included. As the CLASSIC study showed, the improvement in 3-year disease-free survival was not as evident for patients with stage II disease as it was for those with stage III disease in the adjuvant chemotherapy group. In the JCOG8801 study [17], patients with T1-2N0/+ disease were considered to gain no survival benefits from adjuvant chemotherapy. Therefore, whether stage II gastric cancer patients need adjuvant chemotherapy and what kind of patients can benefit from adjuvant chemotherapy remain to be identified. In this study, we used multicenter data in China to explore this question.

The benefits of adjuvant chemotherapy for stage III gastric cancer has been widely demonstrated. However, these benefits remain controversial for stage II gastric cancer [17–19]. Recently, Yuming Jiang et al. [8] reported some prognostic risk factors and tried to predict the survival benefit of adjuvant chemotherapy for patients with stage II and stage III gastric cancer, but no subgroup analysis was carried out in their study. In addition, although many studies have identified a series of prognostic factors related to gastric cancer [20–22], none of the studies discussed stage II gastric cancer separately. Thus, in our study, we attempted to use some available and typical clinicopathological factors and identify their effects on the survival of stage II gastric cancer patients, with the aim to build a model for selecting patients with stage II gastric cancer who can benefit from adjuvant chemotherapy.

Our study demonstrated that age ≥ 60 years, linitis plastica, and T4 were independent prognostic factors in stage II gastric cancer patients. Contrary to the results of other similar studies [23, 24], N stage was not a prognostic factor in the results of the multivariate analysis. Additionally, patients with lymph node metastasis had better OS (mean OS, 104.76; 95% CI, 100.73 to 108.80) than those without lymph node metastasis (mean OS, 100.08; 95% CI, 94.77 to 105.40). In other studies, patients classified as stage II/III or all stages were included. However, stage II (according to 8th edition AJCC guidelines) gastric cancer is a special stage. Stage II gastric cancer patients with a late T stage usually have early N stage disease. Our results confirmed that T stage is more meaningful than N stage in influencing the prognosis of stage II gastric cancer patients. Therefore, due to the late T stage, patients without lymph node metastasis may have a poorer OS than those with lymph node metastasis.

In clinical practice, the TNM staging system is an essential way to evaluate the prognosis of gastric patients [25]. However, we found that the prognosis often varies even within the same TNM stage. As Warneke stated [26], the TNM staging system is a simple mathematical model, and it is difficult to reflect the actual OS without other clinicopathological factors. To assess the prognosis more accurately, other important clinicopathological factors should also be taken into account. As our univariate analysis results showed, age, tumor location, tumor size, and CEA are other factors associated with OS that should be considered. In the past, some studies used similar prognostic risk factors to distinguish patients who can benefit from adjuvant chemotherapy. However, some vital clinicopathological factors were unbalanced, or the number of patients from a single center was relatively small [18, 27].

There is no denying that age must be a key factor in prognosis. Research in the Netherlands has confirmed this association [28]. Toru Aoyama et al. [29] and Pompiliu Piso et al. [30] reported that the long-term survival of patients with distal tumors was more satisfactory than that of patients with proximal tumors. There is a high proportion of undifferentiated tumors in the proximal stomach, which tend to have a poorer prognosis than differentiated tumors. Anatomically, the intra-abdominal part of the cardia and fundus are not fully covered by visceral peritoneum, so proximal gastric cancer is more likely to infiltrate the serosa and more prone to peritoneal metastasis [27]. In terms of surgery, radical surgery for proximal gastric cancer is D2 total gastrectomy, which is more invasive than surgery for distal gastric cancer [29]. Linitis plastica has the poorest prognosis among all tumor locations. In Asia, linitis plastica is defined as Borrmann IV gastric cancer [16]. A recent study showed that linitis plastica has a high risk for peritoneal involvement (75.2% among patients with radiographically nonmetastatic disease), so linitis plastica was an independent prognostic factor [31]. In the past, some studies showed that a larger tumor size may increase the difficulty of surgery or be associated with a more advanced Borrmann type, deeper depth of invasion, and higher incidence of lymph node metastases, all of which contribute to poorer OS [32, 33].

There are some limitations in our study. First, this study is a retrospective study, so selection bias is inevitable. However, we used PSM to adjust the baseline characteristics and reduce the influence of selection bias. Second, considering the large number of cases from multiple centers in this study, our results may be generalized, at least in Asia, but these results need to be further validated in European and American cohorts because all patients in this study were from China. Third, the adjuvant chemotherapy regimens were not uniform, but all the patients who were included in adjuvant chemotherapy group accepted the fluorouracil-based adjuvant chemotherapy. Recently, fluorouracil is still the basis of the adjuvant chemotherapy regimens for gastric cancer (containing XELOX、SOX or S-1). Therefore, it may be reasonable to consider that the effect of different chemotherapy regimens on outcomes in our study would be slight.

## Conclusion

In conclusion, age ≥ 60, linitis plastica and T4 are independent risk prognostic factors, thus patients with these risk factors may need to receive adjuvant chemotherapy. However, adjuvant chemotherapy may be dispensable for other patients with good prognosis in stage II gastric cancer.

## Data Availability

The data used and analysed during this study are available from the corresponding author on reasonable request.
